# TcdC Does Not Significantly Repress Toxin Expression in *Clostridium difficile* 630ΔErm

**DOI:** 10.1371/journal.pone.0043247

**Published:** 2012-08-17

**Authors:** Dennis Bakker, Wiep Klaas Smits, Ed J. Kuijper, Jeroen Corver

**Affiliations:** Department of Medical Microbiology, Center of Infectious Diseases, Leiden University Medical Center, The Netherlands; Institute Pasteur, France

## Abstract

In the past decade, *Clostridium difficile* has emerged as an important gut pathogen. Symptoms of *C. difficile* infection range from mild diarrhea to pseudomembranous colitis, sometimes resulting in colectomy or death. The main virulence factors of *C. difficile* are toxin A and toxin B. Besides the genes encoding these toxins (*tcdA* and *tcdB*), the pathogenicity locus (PaLoc) also contains genes encoding a sigma factor (*tcdR*) and a putative anti-sigma factor (*tcdC*). The important role of TcdR as a sigma factor for toxin expression is undisputed, whereas the role of TcdC as an anti-sigma factor, inhibiting toxin expression, is currently the subject of debate. To clarify the role of TcdC in toxin expression, we generated an isogenic ClosTron-based mutant of *tcdC* in *Clostridium difficile* strain 630Δ Erm (CT::*tcdC*) and determined the transcription levels of the PaLoc genes and the expression levels of the toxins in the wild type strain and the *tcdC* mutant strain. We found only minor differences in transcription levels of the PaLoc genes between the wild type and CT::*tcdC* strains and total toxin levels did not significantly differ either. These results suggest that in *C. difficile* 630Δerm TcdC is not a major regulator of toxin expression under the conditions tested.

## Introduction

*Clostridium difficile* is an anaerobic, Gram-positive, spore forming rod shaped bacterium that can cause disease with a wide variety of symptoms, ranging from mild diarrhea to severe forms of pseudomembranous colitis [1]–[3]. Since 2004, numerous countries have reported outbreaks in health-care facilities caused by hypervirulent *C. difficile* PCR Ribotype (Type) 027 [1]–[6]. *Clostridium difficile* infection (CDI) caused by Type 027 is associated with a more severe course of the disease and a higher mortality rate than other ribotypes [1], [3], [6]. Recently, increasing numbers of the hypervirulent Type 078 are reported [7]. *C. difficile* Type 078 is more frequently associated with community acquired CDI and affects a younger population than Type 027 [6]–[9]. Furthermore, CDI caused by Type 078 is associated with an increased morbidity compared to other ribotypes [8].

The main virulence factors of the enteropathogenic *C. difficile* are the two large clostridial toxins, toxin A (TcdA) and toxin B (TcdB). These toxins are glycosyltransferases that inactivate Rho, Rac and Cdc42, thereby disrupting the cytoskeleton and tight junctions of the cells, resulting in apoptosis [10]. This induces an inflammatory response and degradation of the intestinal epithelial cell layer. Besides the genes encoding these toxins (*tcdA* and *tcdB*), the pathogenicity locus (PaLoc) also contains genes encoding a sigma factor (*tcdR*) and a putative anti-sigma factor (*tcdC*) [11]–[13]. In between the toxin genes the *tcdE* gene is situated, which encodes a putative holin protein [14]. Interestingly, both hypervirulent Types 027 and 078 have been shown to contain mutations in the *tcdC* gene, encoding the putative negative regulator of toxin gene transcription, and this has been proposed as a possible explanation for their increased virulence [8], [15].

The exponential growth phase of *C. difficile* has been reported to be associated with a high transcription level of the *tcdC* gene and low transcription levels of *tcdR* and the toxin genes, whereas the stationary growth phase is associated with a low transcription level of the *tcdC* gene and high transcription levels of *tcdR* and the toxin genes in strain VPI10463 [16]. The synthesis and secretion of the toxins is increased upon entry into the stationary growth phase [16]–[19]. The decreasing transcription of *tcdC* correlates with diminishing TcdC protein levels in stationary growth phase [16], [20].

TcdR is an alternative sigma factor that positively regulates toxin production [11], [12]. The direct interaction of TcdR and the RNA polymerase core enzyme mediates recognition of the toxin promoters and the *tcdR* promoter [11], [12], [21]. TcdC has been reported to act like an anti-sigma factor for toxin production by destabilizing the TcdR-RNA polymerase core enzyme complex in a way that is not yet fully understood [12].

The reported inverse correlation between the transcription of *tcdC* and the toxin genes and the expression patterns of the corresponding proteins, together with the biochemical data, has led to the prevailing model that TcdC is an important repressor of toxin expression [12], [16], [17], [20]. This model seems to be supported by the finding that the absence of a functional TcdC caused by a frame shift mutation (Δ117 bp) in the *tcdC* gene is linked to a supposed increased toxin production in certain (hyper) virulent strains [15], [22].

Recently, some doubts were raised about the importance of TcdC for regulation of toxin expression on the basis of two findings. First, two studies have found increasing levels of *tcdC* transcription in time that coincide with increasing transcription of the toxin genes and increasing amounts of toxin production [18], [19]. Second, there is a great variability in toxin expression levels among (hyper) virulent strains, even though these generally carry mutations in *tcdC* [15], [18], [19]. Therefore, a minor (or modulatory) role for TcdC in the regulation of toxin expression was proposed [18], [19].

Here, we sought to clarify the role of TcdC in regulation of the toxin production by generating an isogenic *tcdC* mutant (CT::*tcdC*) using the ClosTron technology. We find only minor differences in transcription levels of the PaLoc genes between the wild type and CT::*tcdC* strains and the expressed total toxin levels did not significantly differ, suggesting that the role of TcdC in toxin regulation is not of significance under the conditions tested in *C. difficile* strain 630ΔErm.

## Results

The importance of TcdC for regulation of toxin expression was recently challenged by two studies [18], [19]. It was proposed, based on the increasing transcription levels of the PaLoc genes in time and the variability in toxin expression levels among virulent strains, that TcdC has a minor or modulatory role on toxin expression rather than a major role as previously assumed. In this study we sought to clarify the role of TcdC for toxin expression by generating an isogenic *tcdC* mutant. As toxin gene expression is subject to complex regulation influenced by glucose and cysteine, we performed our experiments in a trypton-yeast (TY) based broth [17], [23]. TY broth does not contain glucose and no cysteine was added. We verified that in TY broth earlier and higher expression of toxins was achieved in comparison to the commonly used Brain Heart Infusion (BHI) broth (data not shown).

### Generation and Characterization of a TcdC Mutant

**Figure 1:**
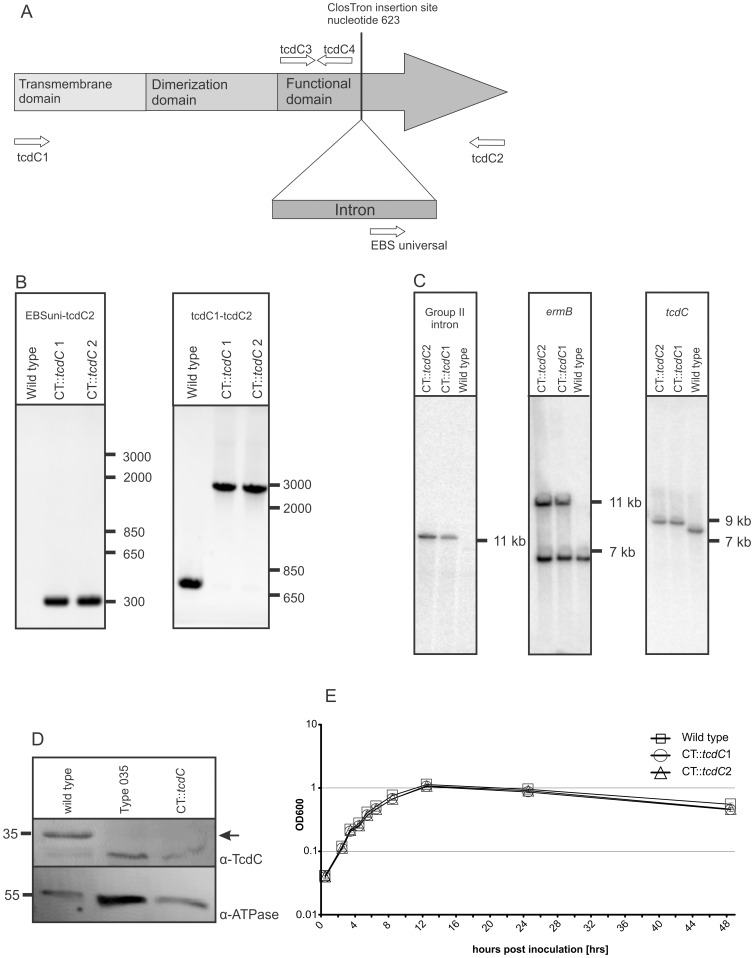
Characterization of the *C. difficile tcdC* mutant. (**A**) Schematic representation of 3 different domains of TcdC and the intron insertion site for the inactivation of TcdC. The arrows in the putative repressor domain represent the locations and orientation of the primers used in the RT-q-PCR and conventional control PCRs. (**B**) PCR confirmation of the wild type strain and the CT::*tcdC* mutant. The primer EBS universal and tcdC2 generated a PCR product of 302 bp for the CT::*tcdC* strains. Primers tcdC1 and tcdC2 generated a 699 bp PCR product for the wild type and for the CT::*tcdC* strain a PCR product of circa 2800 bp. (**C**) Southern blot analysis of EcoRV digested genomic DNA of wild type and CT::*tcdC* strains with a Group II intron, *ermB* gene and *tcdC* specific probes. Note that probing with the *ermB* probe results in 2 bands for the CT::*tcdC* strains, since wild type already carries a copy of the *ermB* gene in the genome [35]. (**D**) Western blot analysis of TcdC production in wild type and CT::*tcdC* strain 8 hours post inoculation. The arrow indicates the location of TcdC protein based on MW and absence of the protein in the PaLoc negative Type 035 strain. Note that cross-reaction of TcdC antibody with a protein of similar MW was also observed in Carter et al. [30]. (**E**) Growth curves of *C. difficile* 630ΔErm and *C. difficile* CT::*tcdC* mutant strains. The absorbance (OD_600_) was measured over 48 hrs of growth in TY medium. The error bars indicate the standard error of the mean of six experiments.

TcdC consists of three domains: a hydrophobic domain, a proposed dimerization domain and a proposed C-terminal repressor domain (Figure 1A) [12]. We successfully disrupted the *tcdC* gene in the region coding for the repressor domain using ClosTron technology. Disruption of genes using the ClosTron technology results in stable mutants and no or non-functional proteins [24]–[26]. The genotype of the disruption was confirmed with conventional PCRs using the tcdC2 primer and the EBS universal primer and with a primer pair (tcdC1 and tcdC2) flanking the ClosTron insertion site (Figure B). Sequence analysis confirmed that the disruption was in the proposed repressor domain of the *tcdC* gene at the expected site (data not shown). In addition, Southern blot analysis using intron-, *ermB* and *tcdC*- specific probes clearly confirmed a specific single insertion of the Group II intron in the genome (Figure 1C).

Western blot analysis, using antibodies against TcdC, confirmed that the isogenic *tcdC* mutant no longer expressed TcdC (Figure 1D). A control blot using antibodies against F_0_F_1_ ATPase indicated that the lack of signal in the TcdC Western blot was not a result of lower amounts of proteins loaded in the lanes of PCR ribotype 035 (a PaLoc negative strain) and the *tcdC* mutant.

The growth kinetics of the wild type and CT::*tcdC* strains showed no significant differences in various media tested (Figure 1E and data not shown). In TY broth, which does not contain glucose or added cysteine, the wild type strain and the CT::*tcdC* strains showed an exponential growth phase in the first 8 hours post inoculation and after 12 hours post inoculation both strains entered into the stationary growth phase (Figure 1E). Conventional control PCRs confirmed that the disruption of the *tcdC* gene had remained intact during our growth curves experiments (data not shown).

### Comparable Relative Transcription Levels of PaLoc Genes in Wild Type and CT::*tcdC*

In order to determine the influence of TcdC on the transcription levels of the PaLoc genes we compared the relative transcription levels of the PaLoc genes of wild type and CT::*tcdC* strains by reverse transcriptase quantitative real-time PCR (RT-qPCR). We found comparable transcription levels of all PaLoc genes in wild type and CT::*tcdC* strains.

**Figure 2:**
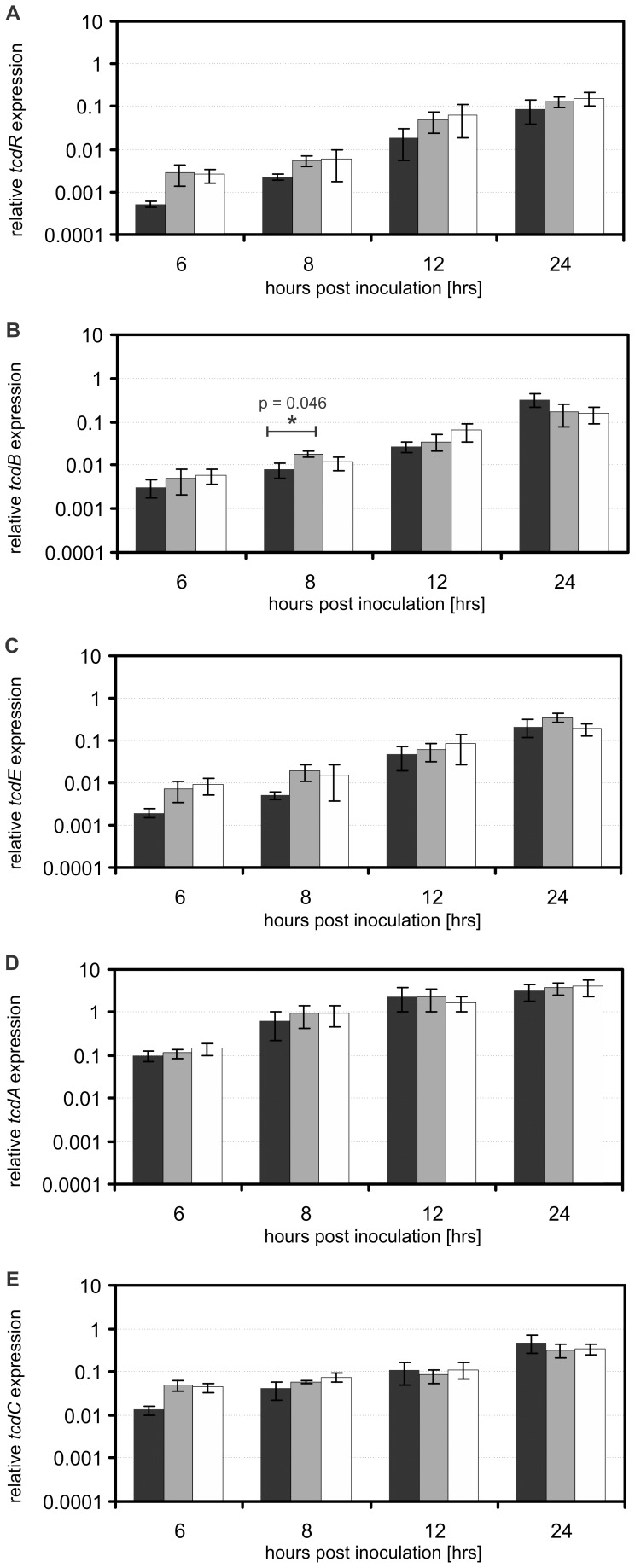
The relative PaLoc gene expression profiles of wild type and CT::*tcdC* in time. The error bars indicate the standard error of the mean (n = 6). The asterisk (*) indicate a significant difference between wild type and CT::*tcdC* strain. Values are normalized to *rpsJ* expression. Wild type corresponds to black bars, CT::*tcdC*1 mutant strains to gray bars, CT::*tcdC*2 to the white bars. (**A**) The relative expression of *tcdR*. (**B**) The relative expression of *tcdB*. (**C**) The relative expression of *tcdE* (**D**) The relative expression of *tcdA*. (**E**) The relative expression of *tcdC*.

Overall, the logarithmic growth phase was associated with lower transcription levels of the PaLoc genes and by entering into the stationary phase increasing transcription levels of PaLoc genes were found, as previously described for *tcdR, tcdE*, *tcdB* and *tcdA* [16], [18], [19] and *tcdC* [18], [19] (Figure 2). The transcription levels of *tcdR* in wild type and CT::*tcdC* strains increased approximately 100-fold between 6 and 24 hours post inoculation (Figure 2A). Though the expression of *tcdR* was, on average, 3-fold higher at the various time points in the CT::*tcdC* strains compared to the wild type, this difference was not statistically significant (Figure 2A, all p values ≥0.088). Similarly, we observed a 10- to 100-fold increase in the transcription levels of *tcdB* (Figure 2B), *tcdE* (Figure 2C), *tcdA* (Figure 2D) and *tcdC* (Figure 2E) when comparing values from the logarithmic growth phase with those observed in the stationary growth phase. The expression levels of *tcdB, tcdE, tcdA and tcdC* were, on average, 1.5-fold, 2.5-fold, 1.4-fold and 1.7-fold higher, respectively, in the CT::*tcdC* strains compared to the wild type. With one exception, these differences were not found to be significant. The transcription level of *tcdB* in the CT::*tcdC*1 strain is significantly (P = 0.046) higher compared to wild type level at 8 hours post inoculation (Figure 2D). However, no significant differences are found between the wild type and CT::*tcdC* strains at any of the other time points.

Therefore, we conclude that the disruption of the *tcdC* gene does not result in a consistently and significantly increased transcription level of the PaLoc genes.

### Comparable Toxin Expression in Wild Type and CT::*tcdC*

Considering the small increase in PaLoc gene expression in the CT::*tcdC* mutants observed in the RT-qPCR experiments, we were interested to see if this difference translated into higher toxin levels. We determined toxin levels using two independent assays, but found no consistent difference between wild type and mutant cells.

**Figure 3:**
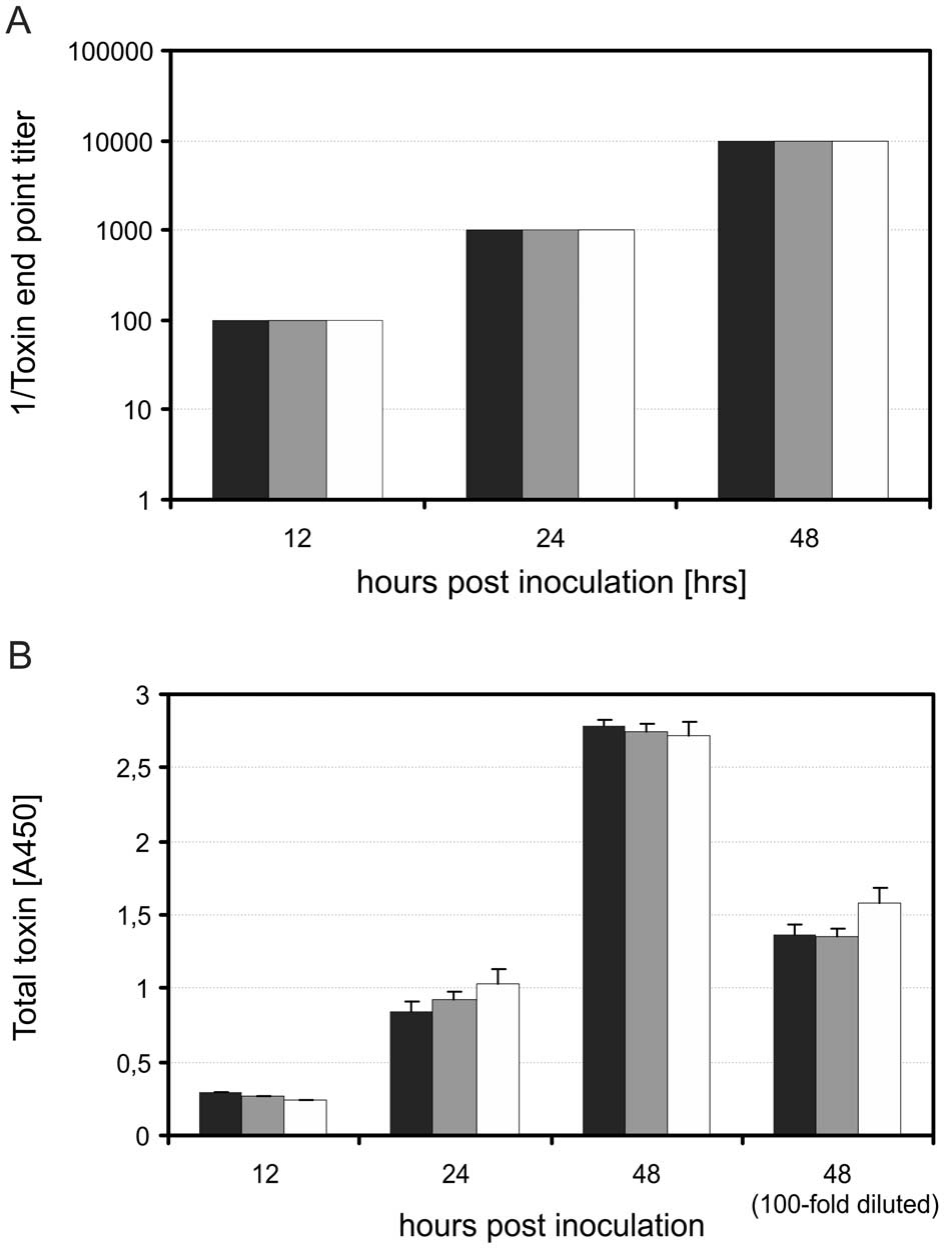
The toxin production profiles of wild type and CT::*tcdC* mutant strains in time. Wild type corresponds to black bars, CT::*tcdC*1 mutant strains to gray bars, CT::*tcdC*2 to the white bars. Total toxin amounts were quantified by using two independent assays. (**A**) The supernatants were incubated in a ten fold dilutions series on Vero cell monolayers. After 24 hrs the cytotoxic effects were quantified by determing the toxin end point titer. Values are given as means (n = 6). (**B**) An enzyme immunoassay was used for direct quantification of the secreted toxins according manufacters protocol. The supernatants of 12 and 24 hours post inoculation were 10 times diluted. The supernatants of 48 hours post inoculation were diluted 10 and 100 times. Values are given as means ± standard error of the mean (n = 6).

First, filter sterilized bacterial supernatants were incubated on a Vero cell (a kind gift of Dr. E.J. Snijder [27]) monolayer and cytotoxic effects were quantified after 24 hours by determining the end-point titer (Figure 3A) [26]. In the exponential growth phase (5 and 8 hours post inoculation) no cytotoxic effects were detectable (data not shown). In the stationary growth phase (12, 24 and 48 hours post inoculation) we observed increasing cytotoxic effects, indicative of the presence of toxin. Importantly, the observed cytotoxic effects were specific for *C. difficile* toxin A and B, as a pre-incubation of the filter sterilized bacterial supernatants with anti-toxin, a polyclonal antibody against toxin A and toxin B (Techlab), resulted in complete neutralization of cytotoxic effects on the Vero cells at all time points (data not shown). The *tcdC* mutant strains showed no significant differences in toxin levels compared to the wild type strain (Fig. 3A).

Next, we used an enzyme immunoassay (Ridascreen, Biopharma) for the direct detection and relative quantification of the secreted toxins. In the exponential growth phase (5 and 8 hours post inoculation) no toxins were detectable (data not shown), consistent with the lack of toxicity towards Vero cells described above. In the stationary growth phase (12, 24 and 48 hours post inoculation) increasing toxin levels were detectable. When we compared the toxin levels at various time points, there were equal amounts of toxins in the wild type and *tcdC* mutant strains.

We conclude that the disruption of the *tcdC* gene does not result in consistently and significantly increased toxin levels.

## Discussion

*C. difficile* infections caused by the (hyper-)virulent Type 027 (NAP1/REA B1) and Type 078 (NAP7/REA BK) are associated with an increased morbidity and severity of disease compared to other types [1], [3]. This increase is suggested to be linked to toxin hyper production [3], [22], [28]. A potential mechanism by which this could occur is through inactivation of a negative regulator of the toxin gene transcription. TcdC has been identified as a negative regulator of toxin production [12].

In the currently prevailing model, a major role for TcdC in the repression of toxin genes has been proposed on the basis of three lines of evidence. First, in *C. difficile* VPI10463 (a high toxin producing strain that also expresses high levels of TcdC [18], [29]), an inverse correlation between the transcription of *tcdC* and the genes encoding the toxins is found [16], [18], [29]. This correlation for TcdC is also observed in protein levels [20]. Second, elegant *in vitro* experiments have established that heterologously produced and purified TcdC protein can interfere with TcdR-mediated transcription of toxin genes in a way that is not yet fully understood [12]. Finally, a frame shift mutation (Δ117 bp) in *tcdC*, that results in a non-functional protein, is associated with increased toxin production in certain (hyper)virulent strains [15], [22].

Recently, it was reported that the introduction of a functional *tcdC* gene from a high toxin-producing strain that lacks any of the hyper virulence associated *tcdC* mutations (VPI10463, PCR ribotype 087) into an epidemic strain carrying a non-functional *tcdC* (M7404, PCR ribotype 027/NAP1/REA B1) can reduce toxin expression levels and moderately attenuate virulence [30]. This data seems consistent with the model discussed above. However, it is unclear how the levels of TcdC in the complemented strain relate to the physiological levels of the protein prior to the inactivation of TcdC in this strain background. The introduced *tcdC* gene, including its transcription signals, was derived from a different genetic background (VPI10463, Type 087) and was introduced on a multicopy plasmid. In addition, the reintroduction of TcdC in a strain lacking a functional TcdC, may affect processes that are not normally affected. Finally, the experiments were not corrected for the additional copies of the *tcdC* promoter that could result in the titration of regulators binding to those sequences.

In an alternative approach that addresses many of the issues above, the role of *tcdC* in toxin expression could be addressed by removing it from a background in which it is normally functional. To this end, we generated two independent isogenic ClosTron-based *tcdC* mutants strain that could be directly compared to its wild type counterpart, in which the TcdC protein was expected to be functional. Our data obtained with these mutant strains show that TcdC does not exert a major or even significant effect on the transcription of the PaLoc genes or the expression levels of the toxins under the conditions tested.

Our experiments were performed in a glucose free TY broth medium, since glucose is a known repressor of toxin production [17]. Indeed, we observed earlier and higher levels of toxin production in TY broth than in the commonly used Brain-Heat-Infusion broth (BHIS) based media, that does contain low amounts of glucose (0.2%) and to which frequently cysteine is added. However, also in BHIS we did not observe a significant effect of a *tcdC* deletion on toxin expression (data not shown).

We controlled critical parameters in our experiments by performing conventional PCRs which confirmed that the disruption of *tcdC* remained intact throughout the growth curve. Western blot analysis with antibodies raised against a TcdC epitope confirmed that the disruption of the *tcdC* gene resulted in the absence of TcdC protein (Figure 1D). The disruption of the *tcdC* gene did not affect the growth kinetics compared to the wild type strain (Figure 1E).

In the RT-qPCR experiments, sample to sample variation was corrected by normalizing to the reference gene *rpsJ* [31]. The *rpsJ* gene was selected for normalization, since *rpsJ* was overall the highest ranked reference gene regarding gene expression stability [31].Reverse transcription was carried out using random hexamers, to prevent gene specific biases [32]. PCR efficiency in the qPCR was determined using a standard curve for each gene, enabling post run correction [33]. To obtain objective data concerning the quantification of the secreted toxins, we used an end point titer assay and an enzyme immunoassay rather than a manual (subjective) cell scoring system [26].

The trends observed in the transcription of the PaLoc genes and the expression of the toxins generally conform to previously reported data [18], [19]. It should be noted that the up-regulation in time of *tcdC* transcription was not observed in earlier studies on *C. difficile* VPI10463 [16] but is consistent with more recent reports [18], [19]. We observed an increase in transcription of the PaLoc genes in time, and a concomitant increase in toxicity of culture supernatant in stationary phase that can be attributed to the toxins as it is fully neutralized by anti-toxin against toxin A and B.

The disruption of the *tcdC* gene resulted in an on average 1.7 fold higher transcription level of *tcdC* in time compared to the wild type strain, although this difference was not found to be statistically significant. It should be noted that we detect these differences because the real time PCR probe detects a region of the gene upstream of the ClosTron insertion site (Figure 1A). This finding might indicate some kind of feedback mechanism on TcdC expression. Similar to *tcdC* gene expression, the disruption of *tcdC* resulted in a slightly higher transcription level of the other PaLoc genes, although this was generally not significant. Moreover, the increased transcription level of the toxin genes did not result in a detectable increase in toxin levels as measured with two independent assays.

Based on the paradigm that TcdC is a major suppressor of toxin production we expected precocious and significantly elevated transcription levels of *tcdA*, *tcdB, tcdE* and *tcdR* in the CT::*tcdC* strains compared to wild type. However, our data indicate that TcdC exerts a moderate, if any, effect on the transcriptional levels of the PaLoc genes and the expression of toxins in *C. difficile* 630Δerm under the conditions tested.

*Clostridium difficile* strain 630ΔErm is a derivative of the clinical isolate 630 [34], [35], a PCR ribotype 012 strain. PCR ribotypes 012 strains constitute 4% of the clinically isolated toxinogenic isolates in Europe [7]. *Clostridium difficile* 630 (PCR ribotype 012)-derived strains are commonly used to investigate virulence of mutants [26], [36], [37].

An independent study, published during the preparation of this manuscript, reached a similar conclusion with respect to the role of TcdC in toxin regulation in *C. difficile* 630Δerm using an allelic exchange technique [38]. In that paper reintroduction of a single functional copy of *tcdC* at its native locus did not affect toxin production in strain R20291 either [38]. R20291 is a strain from problematic PCR ribotype 027 (NAP1/REA B1) that was isolated following an outbreak in Stoke Mandeville, UK.

Our work and that of Cartman and coworkers [38] seem at odds with the previous reports that clearly demonstrate that TcdC can act as a repressor for toxin gene expression [12], [30]. However, we cannot exclude the possibility that TcdC exerts a more profound effect under specific conditions, or in other strains of *C. difficile* than 630Δerm and R20291. It should be clear though that *in vivo* relevance of TcdC for toxin regulation in these two strains is limited.

In conclusion, we suggest that TcdC might have a modulatory role in regulating toxin expression, and that TcdC functionality is therefore not a major determinant of the (hyper)virulence of *C. difficile*. This is supported by the lack of correlation between virulence, toxin production and *tcdC* gene variants that was noted by several other studies [18], [19], [30], [39].

## Materials and Methods

### Bacterial Strains and Growth Conditions

The *Clostridium difficile* and *Escherichia coli* (*E. coli*) strains and plasmids used in this study are described in Table 1. *E. coli* strains were grown in Luria Bertani (LB, USB cooperation) medium supplemented with appropriate antibiotics when required. *C. difficile* strains were grown anaerobically in a microaerobic cabinet (Don Whitley VA1000) at 37°C in pre-reduced 3% Bacto Tryptose (Difco), 2% Yeast extract (Difco) and 0.1% thioglycolate (pH 7.4) medium (TY) or Brain Heart Infusion broth (Oxoid) supplemented with 0.5% yeast extract and 0.01% L-cysteine (Sigma) (BHIS) [40], [41]. When required, the broths were supplemented with appropriated antibiotics. For RNA extraction and toxin quantification, *C. difficile* 630ΔErm (wild type) and two independent isogenic *tcdC* mutant strains (CT::*tcdC*) were serially diluted and pre-cultured (overnight) in pre-reduced TY broth. Mid-logarithmic growth phase pre-cultures (OD_600_ 0.4–0.8) were used to inoculate pre-reduced TY broth to a starting OD_600_ of 0.05 (±0.01). Optical density readings and samples for total toxin quantification were taken at 2, 3, 4, 5, 6 and 8 hours post inoculation in the exponential growth phase and at 12, 24 and 48 hours post inoculation in the stationary phase. Samples for RNA extraction were taken at 6, 8, 12, and 24 hours post inoculation. Samples for Western blot detection of TcdC were taken at 8 hours post inoculation. We routinely monitored the purity of the *C. difficile* cultures by culturing on appropriate agar plates and performed control PCRs to ensure that the insertional disruption of the *tcdC* gene had remained intact during our experiments. All experiments were performed six times.

**Table 1:** Strains and plasmids used in this study.

Strains	Description	Origin
***Escheria coli***		
DH5α	Erythromycin^S^, Lincomycin^S^	Laboratory stock
CA434	Erythromycin^S^, Lincomycin^S^, Kanamycin^R^, plasmid R702	[41]
***Clostridium difficile***		
630ΔErm (wt)	Erythromycin^s^, Lincomycin^S^	[34]
Leeds_ 035	Type 035, *tcdC* negative, PaLoc negative	[44]
*CT::tcdC*1	630ΔErmΔtcdC623as, Erythromycin^R^, Lincomycin^R^	This study
*CT::tcdC*2	630ΔErmΔtcdC623as, Erythromycin^R^, Lincomycin^R^	This study
**Plasmids**		
pMTL007C-E2	Thiamphenicol^R^, Erythromycin^S^	[25]
pDB001AAATTAGAAACTTGCGTTCAGTAAACE2:*tcdC*623as	pMTL007C- E2:*tcdC*623as	This study

### Generation of *tcdC* Mutant Strains

We generated two independent isogenic *tcdC* mutants by insertional inactivation of the *tcdC* gene in the wild type strain 630Δerm using ClosTron technology [24], [25]. Briefly, the Perutka algorithm on the ClosTron website (http://www.clostron.com) was used to design primers (Table 2) for retargeting the Group II intron (Sigma; Targetron). The retargeted intron was cloned using the restriction enzymes BsrGI and HindIII into plasmids pMTL007C-E2 and the constructs were verified by sequencing [25]. The verified plasmid (pDB001) was transformed to *E. coli* CA434 and transferred to the wild type strain 630Δerm via conjugation [34], [41]. The selection of *C. difficile* transconjugants was done by subculturing on pre-reduced BHIS agar supplemented with thiamphenicol (Sigma; 10 µg/ml) and *C. difficile* selective supplement (Oxoid). This was followed by several rounds of subculturing on pre-reduced BHIS agar supplemented with lincomycin (Sigma; 20 µg/ml) and *C. difficile* selective supplement to promote integration of the GroupII intron into the gene of interest. Chromosomal DNA isolated from the transconjugants using a QIAamp blood kit (Qiagen) was used in conventional PCRs and sequence runs to confirm the disruption of *tcdC* and the nucleotide position of the insertion in the *tcdC* gene. Primers used for cloning and sequencing are listed in Table 2.

**Table 2:** Primers and probes used in this study.

Primers		
IBS-tcdC623as	AAAAAAGCTTATAATTATCCTTAGTTATCGTTCCAGTGCGCCCAGATAGGGTG	This study
EBS2-tcdC623as	TGAACGCAAGTTTCTAATTTCGATTATAACTCGATAGAGGAAAGTGTCT	This study
EBS1d-tcdC623as	CAGATTGTACAAATGTGGTGATAACAGATAAGTCGTTCCAGCTAACTTACCTTTCTTTGT	This study
EBS universal	Intron mutagenesis/Control PCR/CGAAATTAGAAACTTGCGTTCAGTAAAC TGAACGCAAGTTTCTAATTTCGATTATAACTCGATAGAGGAAAGTGTCT	[25]
*tcdC*1	Control PCR/ATGTTTTCTAAAAAAAATGAT	This study
*tcdC*2	Control PCR/TTAATTAATTTTCTCTACAGCT	This study
*tcdR* Forward	Multiplex 1/ATAATGATGCCCACAAGATGATTTAG	This study
*tcdR* Reverse	Multiplex 1/AAAGAAGTGATCTATATCATCAGTTAC	This study
*tcdR* probe	Multiplex 1/TEX-TATGACCTGAACCACCTTCCATTCTCC-BHQ-2	This study
*tcdB* forward	Multiplex 1/ATAATGATGCCCACAAGATGATTTAG	This study
*tcdB* Reverse	Multiplex 1/AAAGAAGTGATCTATATCATCAGTTAC	This study
*tcdB* probe	Multiplex 1/TEX-TATGACCTGAACCACCTTCCATTCTCC-BHQ-2	This study
*tcdE* Forward	Multiplex 2/ATTTGATACATTATTAGGATGTTTAAG	This study
*tcdE* Reverse	Multiplex 2/AAATATACATGCTATCATTGCTAC	This study
*tcdE* probe	Multiplex 2/FAM-TGATTCCTCCATCTATTCCAAAACTAGAA-BHQ-1	This study
*tcdA* forward	Multiplex 1/AATTCCAATACAAGCCCTGTAG	This study
*tcdA* Reverse	Multiplex 1/TATCAGCCCATTGTTTTATGTATTC	This study
*tcdA* probe	Multiplex 1/FAM-ATCACTGACTTCTCCACCTATCCATACAA-BHQ-1	This study
*tcdC*3	Multiplex 1/CATAATTTCCAGACACAGCTAATC	This study
*tcdC*4	Multiplex 1/GGATATGATACTGGTATTACTTATGAC	This study
*tcdC* probe	Multiplex 1/YAK-TGCACCTCATCACCATCTTCAATAACTTG-BHQ1	This study
*rspJ* Forward	GATCACAAGTTTCAGGACCTG	This study
*rspJ* Reverse	GTCTTAGGTGTTGGATTAGC	This study
*tcdC5*	CATATCCTTTCTTCTCCTCTTC	This study
*tcdC6*	AATTGTCTGATGCTGAACC	This study
oWKS-1131	AAAGCGATGCCGAGAATCTG	This study
oWKS-1132	TCTCGGAGTATACGGCTCTG	This study
Sal-R1	ATTACTGTGACTGGTTTGCACCACCCTCTTCG	[45]

Complementation can be a valuable control for knockout studies. However, as our *tcdC* mutant strains have no clearly detectable phenotype regarding toxin production, complemented mutant strains are expected to be comparable to wild type and *tcdC* mutant strains, as also reported recently in an independent study [38]. Therefore, a complementation study would not add to the message of this manuscript.

### Southern Blots

Southern blot analysis was performed to verify a specific single integration into the genome. Genomic DNA was extracted using a Phenol-chloroform extraction [42]. Four µg of genomic DNA was digested with EcoRV enzyme and separated on a 0.8% agarose/0.5xTris-acetate-EDTA gel by electrophoresis. DNA was transferred onto a Hybond N+ filter (Amersham) in 10x saline sodium citrate (SSC) solution. The filter was washed in 2X SSC and baked at 80°C for 2 hours. Prehybridization of the filter was done for 2 hrs at 60°C in 5x SCC, 5x Denhart and 100 mg/ml of yeast tRNA. Probes specific for the group II intron (EBS2-tcdC623as/Sal-R1), *ermB* gene (oWKS1131/oWKS1132) and *tcdC* gene (tcdC5-tcdC6) were generated. Primers are listed in Table 2. The generated probes (100 ng) were radiolabeled (^32^P dATP) using Klenow enzyme (Roche) and overnight hybridized in 10 ml fresh pre-hybridization buffer at 60°C. The filter was washed for 30 min in 2x SCC, 0.5% SDS, 30 min in 1X SSC, 0.5% SDS and 30 min in 0.5X SSC, 0.5% SDS and analyzed using phosphorimage screen and a Typhoon 9410 scanner (GE healthcare).

### Western Blots

Antibodies against TcdC were generated by immunizing rabbits with a synthetic peptide (CQLARTPDDYKYKKV) representing a specific TcdC epitope (Genscript). Note that this epitope is located before the Clostron insertion site, and would therefore also be expected to detect truncated TcdC protein, would this be produced. Western blots were performed as follows. *C. difficile* (2 mL) cultures were harvested by centrifugation (2 min, 11.000×g, 4°C) and washed with Phosphate Buffered Saline (PBS). The bacterial pellets were resuspended in PBS containing protease inhibitor cocktail (Complete, Roche) and lysed by sonification. The bacterial lysates were centrifuged at low speed (3 min, 1000×g, 4°C) to remove unbroken bacterial cells [20]. To separate the cytosolic proteins from the membrane associated proteins the bacterial supernatant was centrifuged at 200.000×g, 4°C for 1 hr [20]. The pelleted membrane associated proteins were resuspended in 10 mM Tris-HCl (pH 7.4), 5 mM EDTA with 2% Triton X-100 for 30 min at room temperature. Equal amounts of the resuspended membrane associated proteins were separated on 15% SDS PAGE gel and transferred onto polyvinyl difluoride (PVDF) membranes. Similarly generated membranes with the transferred membrane associated proteins of a Type 035 (PaLoc negative) strain were used for pre incubation of the TcdC antibodies. The membranes were probed with the pre incubated TcdC antibody and an antibody against the β subunit of the *E. coli* F_0_F_1_ ATPase that cross reacts with the homologous protein in *C. difficile* [20], [43]. The probed membranes were analyzed using secondary anti-mouse horse radish peroxidase conjugated antibodies (Dako), a chemiluminescence detection kit (Amersham) and a Typhoon 9410 scanner (GE Healthcare).

### RNA Extraction

Five mL of the *C. difficile* cultures were 1∶1 diluted with ice cold methanol and stored overnight at −80°C. Bacterial pellets, obtained by centrifugation (20 min, 3000×g, 4°C), were resuspended into 200 µl lysisbuffer (100 mM EDTA, 200 mM Tris-HCl pH 7.0, 50 mg/ml lysozyme) and incubated for 1 hr at 37°C. Tri-pure reagent (Roche) was used for the extraction of RNA according to the manufacturer’s instruction with minor modifications. Briefly, 1 ml Tri-pure was added to the lysed bacterial pellets and incubated for 5 min at room temperature. Per 1 ml Tri-pure, 200 µl chloroform was added and carefully shaken by hand for 3 min, followed by an incubation of 2–5 min at room temperature. The aqueous phase was collected after centrifugation (12,000×g for 15 min at 4°C) and transferred to a fresh tube. RNA was precipitated by mixing the aqueous phase with 500 µl isopropanol, followed by an incubation of 10 min at room temperature. The precipitated RNA was collected by centrifugation (12,000×g, 10 min, 4°C) and resuspended in 100 µl DNase/RNase free water. The RNA was re-precipitated overnight at −80°C with ammonium acetate (Fluka; 10 mM) and 3 volumes of absolute ethanol. The re-precipitated RNA was washed once with 80% ethanol and dissolved in 50 µl DNase/RNase free water. The RNA was treated twice with a TurboDNase (Ambion) according to the manufacturer’s instruction followed by another Tri-pure RNA isolation. The quality and purity of the extracted RNA was assessed using a RNA nano chip on an Agilent Bioanalyzer.

### Transcriptional Analysis of the PaLoc Genes

A RevertAid™ H Minus Reverse Transcriptase kit (Fermentas) was used to synthesize cDNA according to the manufacturer’s instruction. Random hexamers were used to convert 750 ng RNA into cDNA. The synthesized cDNA was treated with RNase (Qiagen) for 1 hour at 37°C and stored at −20°C. The software program Molecular Beacon (Premier Biosoft) was used to design primer pairs and probes (Table 2) for the 2 multiplex quantitative PCRs (qPCR), based on the available genome of *C. difficile* strain 630 [35]. All primer pairs were first tested by conventional PCR and multiplex PCR to confirm specificity and amplicon sizes. The primer pair and the probe for the amplification of the *tcdC* gene are in front of the insertion site in the *tcdC* gene (Figure 1A), allowing detection of *tcdC* transcription levels in wild type and CT::*tcdC* strains. The real-time multiplex qPCR amplification of the PaLoc genes and the reference gene encoding for a ribosomal protein (*rpsJ*) was performed on a CFX96 real-time PCR detection system (Biorad) [31]. The amplification efficiencies of the PaLoc and reference genes were determined using serially diluted genomic DNA (standard curve). The manually calculated efficiencies and the reference gene *rpsJ* were used to normalize the expression levels of the PaLoc genes. The amplification was performed in a 25 µl final volume. The first real-time multiplex qPCR (target genes: *tcdA*, *tcdA* and *tcdC*) contained 25 µl Hotstar mastermix (Qiagen), forward and reverse primers (80 nm each primer), 2.5 mM MgCl_2_, 100 nM of each probe and 2 µl synthesized cDNA. The second multiplex real-time multiplex Q-PCR (target genes: *tcdR*, *tcdE*) contained 25 µl Hotstar mastermix (Qiagen), forward and reverse primers (80 nm each primer), 3.5 mM MgCl_2_, 100 nM of each probe and 2 µl synthesized cDNA. The real-time qPCR to quantify the reference gene *rpsJ* contained 25 µl Hotstar mastermix (Qiagen), forward and reverse primers (80 nm each primer), 2.5 mM MgCl_2_, 0.06% SYBRgreen (Sigma) and 2 µl synthesized cDNA. The real-time qPCR protocol included an enzyme activation step for 15 min at 95°C, followed by 50 cycles of amplification; 95°C for 30 sec, 52°C for 30 sec and 72°C for 30 sec.

### Relative Quantification of Toxin Expression

Total toxin amounts were quantified using 2 assays; a toxin end point titer assay and a commercial available ELISA (Ridascreen, Biopharma). The supernatants of culture samples (1 mL) were collected after centrifugation (30 min, 3000×g, 4°C), filter sterilized (0.45 µM cellulose acetate membrane) and stored at 4°C.

For the toxin end point titer assay, Vero cells were seeded into a 96 wells plate at a density of 1×10^4^ cells per well and incubated overnight at 37°C and 5% CO_2_. The filter sterilized supernatants of 5, 8, 12, 24 and 48 hours post inoculation (hpi) were diluted 2, 10^1^, 10^2^, 10^3^, 10^4^ and 10^5^ fold in cell culture medium (Dulbecco modified Eagle medium (Lonza) supplemented with penicillin 100 u/mL, streptomycin 100 U/mL, fetal calf serum(10%). Fifty µl of the dilutions were added onto the Vero cell monolayers and incubated for 1 hr at 37°C and 5% CO_2_. For the neutralization assay a 2-fold dilution of each tested time point (5, 8, 12, 24 and 48 hpi) was pre-incubated with a 1/100 diluted anti-toxin (Techlab) for 1 hr at 37°C and 5% CO_2_. After the pre-incubation, 50 µl was added onto the Vero cell monolayers. The incubated bacterial supernatants were aspirated off after one hour and replaced with 100 µl cell culture medium. After 24 hrs of incubation the end-point titer was determined of each diluted time point [26]. The end-point titer was defined as the first dilution at which the Vero Cell morphology was indistinguishable from the neutralized 2-fold diluted supernatants [26]. The enzyme immunoassay (Ridascreen, Biopharma) was performed according manufacture’s protocol.

### Statistical Analysis

Statistical analysis was performed using the software package SPSS 18 (IBM). An independent sample t-test was employed to compare the strains at different time points.
